# Supplementary material for: Long-baseline quantum sensor network as dark matter haloscope
Source: Nat Commun. 2024 Apr 18;15:3331. doi: 10.1038/s41467-024-47566-0 (PMC11026481; doi:10.1038/s41467-024-47566-0)
Supplement: Supplementary file 1 — Supplementary Information [file 41467_2024_47566_MOESM1_ESM.pdf]

# Supplementary Information for: "Long-Baseline Quantum Sensor Network as Dark Matter Haloscope"

Min Jiang,[1,2,3,*] Taizhou Hong,[1,2,3,*] Dongdong Hu,[4,*] Yifan Chen,[5] Fengwei Yang,[6] Tao Hu,[7] Xiaodong Yang,[7] Jing Shu,[8,9,10,†] Yue Zhao,[6,‡] Xinhua Peng,[1,2,3,§] and Jiangfeng Du[1,2,3,11]

[1]*CAS Key Laboratory of Microscale Magnetic Resonance and School of Physical Sciences, University of Science and Technology of China, Hefei, Anhui 230026, China*
[2]*CAS Center for Excellence in Quantum Information and Quantum Physics, University of Science and Technology of China, Hefei, Anhui 230026, China*
[3]*Hefei National Laboratory, University of Science and Technology of China, Hefei 230088, China*
[4]*State Key Laboratory of Particle Detection and Electronics, University of Science and Technology of China, Hefei, Anhui 230026, China*
[5]*Niels Bohr International Academy, Niels Bohr Institute, Blegdamsvej 17, Copenhagen 2100, Denmark*
[6]*Department of Physics and Astronomy, University of Utah, Salt Lake City, Utah 84112, USA*
[7]*Suzhou Institute of Biomedical Engineering and Technology Chinese Academy of Sciences, Suzhou, Jiangsu 215163, China*
[8]*School of Physics and State Key Laboratory of Nuclear Physics and Technology, Peking University, Beijing 100871, China*
[9]*Center for High Energy Physics, Peking University, Beijing 100871, China*
[10]*Beijing Laser Acceleration Innovation Center, Huairou, Beijing, 101400, China*
[11]*Institute of Quantum Sensing and School of Physics, Zhejiang University, Hangzhou 310027, China*

(Dated: March 15, 2024)

**CONTENTS**

I. Dark photon electrodynamics 2

II. Detection efficiency of dark photon 3

III. Quantum sensor network 4

IV. Network statistics 5
A. Signal-to-noise ratio 5
B. DPDM signal and its power 7
C. The connection between SNR and DPDM-induced magnetic field 8

V. Common-mode noise discrimination 8

VI. Data analysis 9
A. Search experiment 9
B. Calculating SNR distribution 10
C. Detection threshold determination 10
D. Exclusion of potential DPDM candidates 11
E. Constraints on dark-photon kinetic mixing coefficient 11

VII. Testing the data analysis procedure with simulated DPDM signals 12

References 12

* These authors contributed equally to this work
† jshu@pku.edu.cn
‡ zhaoyue@physics.utah.edu
§ xhpeng@ustc.edu.cn

## I. DARK PHOTON ELECTRODYNAMICS

This section presents the details of dark photon electrodynamics and derives the magnetic-field signal produced by dark photon dark matter (DPDM). We first consider the dark photon electrodynamics describing a massive U(1) vector boson that kinetically mixes with the electromagnetic photon. Here we choose the interaction basis to describe the Lagrangian [1, 2]

$$\mathscr{L} = -\frac{1}{4}\left(F_{\mu\nu}F^{\mu\nu} + F'_{\mu\nu}F'^{\mu\nu}\right) + \frac{1}{2}m_{A'}^2 A'_\mu A'^\mu - A_\mu\left(eJ^\mu_{\rm EM} - \varepsilon m_{A'}^2 A'^\mu\right), \tag{S1}$$

where $A_\mu$ and $F_{\mu\nu}$ ($F^{\mu\nu}$) are the gauge potential and field strength of electromagnetic field, $A'_\mu$, $F'_{\mu\nu}$ ($F'^{\mu\nu}$) are the ones of dark photon, $m_{A'}$ is the dark-photon mass, $J^\mu_{\rm EM}$ is the electromagnetic current, and $\varepsilon$ is the kinetic mixing coefficient. Here $\varepsilon$ is the important but unknown parameter to be measured in this work. According to Eq. S1, the "$-\varepsilon m_{A'}^2 A'^\mu$" is similar to the electromagnetic current $eJ^\mu_{\rm EM}$. As a result, the dark photon can generate an effective electromagnetic current with components

$$J^\mu_{\rm eff} = -\varepsilon m_{A'}^2 A'^\mu. \tag{S2}$$

When the dark photon has small mass and kinetic mixing coefficient $\varepsilon$, it behaves as noninteracting, stable, cold matter. Therefore, the dark photon can be a good dark matter candidate. When dark photon is the dominant dark matter with $m_{A'}$ below $O(1)$ eV, it behaves as a classical field with an oscillation frequency $\omega \approx m_{A'}$ and bandwidth $\Delta\omega \approx 10^{-6} m_{A'}$ due to the virilization. Thus the effective current in Eq. S2 is an alternating current (AC) source. For example, the dark-photon mass range searched in our work is $4.14\times10^{-15}$ eV to $2.07\times10^{-12}$ eV, corresponding to dark-photon oscillation frequency from 1-500 Hz.

In order to estimate the amplitude of DPDM induced effective electromagnetic current, we should know the gauge potential $A'^\mu$. In the unitary gauge, the 0 component of $A'^\mu$ is sub-leading compared to the spatial part $\vec{\mathbf{A}}'$. The strength of the current $\vec{\mathbf{J}}_{\rm eff} = -\varepsilon m_{A'}^2 \vec{\mathbf{A}}'$ can be estimated by the Galactic dark-matter energy density [3–5]

$$\rho = \frac{1}{2}m_{A'}^2 |\vec{\mathbf{A}}'^2| \approx 0.45\,\mathrm{GeV/cm^3}. \tag{S3}$$

However, the Galactic dark-matter energy density only provides the value of $|\vec{\mathbf{A}}'|$, instead of its three spatial components. For randomized dark photon dark matter profile considered in this work, the three perpendicular projection of the DPDM wavefunction share the same average value, i.e., $\langle {A'_x}^2\rangle = \langle {A'_y}^2\rangle = \langle {A'_z}^2\rangle \approx \sqrt{2\rho/3}/m_{A'}$.

In order to measure the DPDM-induced effective current, we use a conducting shield to localize the excited electromagnetic field within it. These electromagnetic fields satisfy a boundary condition similar to the one of a cavity, forming a basis of cavity modes $\vec{E}_n$. When the dark-photon frequency $\omega$ matches certain mode's resonance frequency $\omega_n$, DPDM resonantly excites the mode with a signal proportional to the overlapping factor $\int_V \vec{E}_n\cdot\vec{\mathbf{J}}_{\rm eff}\,\mathrm{d}V$. In contrast, when the dark-photon frequency $\omega$ is far away from the shield mode's resonance frequencies, only off-resonant modes are excited and dominated by magnetic fields. Our experiment represents the second case. We operate within a dark-photon frequency range of 1-500 Hz, where the corresponding Compton wavelength of the dark photon is much larger than the size of our shield. Generally, the magnetic field has an amplitude

$$B \approx |\vec{\mathbf{J}}_{\rm eff}|\, V^{1/3} \approx 1.63\times10^{-12}\,\varepsilon\left(\frac{m_{A'}}{10\,\mathrm{Hz}}\right)\left(\frac{V^{1/3}}{1\,\mathrm{m}}\right)\mathrm{T}, \tag{S4}$$

where we use the condition that the Galactic dark-matter energy density is $\rho \approx 0.45\,\mathrm{GeV/cm^3}$.

Two rectangular shield rooms located in Harbin and Suzhou, China, with a distance of about 1700 km are exploited to search for DPDM-induced magnetic field. To make the cross correlation of the DPDM-induced magnetic fields at two separate shield rooms as large as possible, we focus on the DPDM wavefunction projection to the $z$-axis (i.e., perpendicular to the Earth's ground surface). This is because the $z$ axes of such two shield rooms are nearly parallel. In this case, the DPDM-induced magnetic field is along the transverse direction (the $xy$ plane). Based on direct electrodynamics calculation, the wavefunctions of the DPDM-induced field follow TM110 modes of a rectangular cavity with

$$B_x^{110} \propto \frac{1}{l_y}\sin\frac{\pi x}{l_x}\cos\frac{\pi y}{l_y}, \qquad B_y^{110} \propto \frac{1}{l_x}\sin\frac{\pi y}{l_y}\cos\frac{\pi x}{l_x}, \tag{S5}$$

where $l_x$ and $l_y$ are the length of the shield room along the $x$ and $y$ direction respectively. The maximum of $B_x$ is at $x = l_x/2$, $y = 0$ or $l_y$, and any value of $z$. The normalization of the DPDM magnetic field can be determined through the overlapping between cavity mode's electric field and the effective current, rendering

$$B_x^{110}(x = l_x/2, y = 0) = \frac{16 l_x^2 l_y}{\pi^3\left(l_x^2 + l_y^2\right)}|\vec{\mathbf{J}}_{\rm eff}|, \qquad B_y^{110}(x = 0, y = l_y/2) = \frac{16 l_y^2 l_x}{\pi^3\left(l_x^2 + l_y^2\right)}|\vec{\mathbf{J}}_{\rm eff}|. \tag{S6}$$

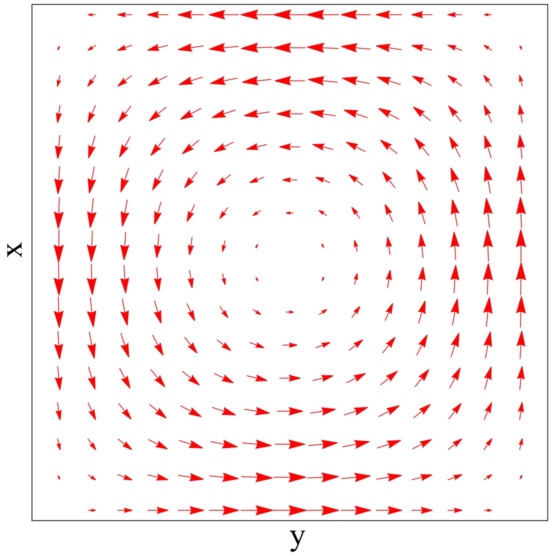


FIG. S1. **Top view of the TM110 mode-magnetic filed**. The dark photon (DP)-induced current $\vec{\mathbf{J}}_{\text{eff}}$ along $z$ produces the magnetic field in the $xy$ plane. Here we focus on the TM110 mode that is dominant component. The amplitude of the DP-induced magnetic field reaches a maximum on the surface of the shield wall.

In addition, higher-order cavity modes also lead to the enhancement of DPDM magnetic field. For $l_x = l_y$ as we considered here, the local magnetic fields from TM130, TM310 and TM330 contributes $\sim$10% of the one from TM110. Taking the size of our shield to be $2 \times 2 \times 2$ m$^3$, one gets a factor of $\approx 0.57$ m for $V^{1/3}$.

According to Eq. S6, we note that a large spatial size of the shield can enhance the DPDM-induced magnetic field. This provides an important approach to improving the DPDM signal. In our experiment, both shield rooms are made of five-layer mu-metal and their innermost layer is cuboid in shape with the dimension of $2 \times 2 \times 2$ m$^3$, which is much larger than that of the shield usually used in previous dark-matter experiments, where their innermost shields' size usually are on the order of 10 cm.

Our experimental configurations measure the dark photon wavefunctions projected along the vertical axis. As a result, certain factors, such as the Earth's rotation over the entire measurement period and the angular separation between the Harbin and Suzhou sites, may have a minor impact on the dark photon-dark matter signals. In the following, we will assess the extent of this influence on the DPDM signals. The two sites, Harbin and Suzhou, are separated by approximately 1400 km. The angular difference along the vertical axis between these locations is roughly $1400/R_E \approx 0.22$, where $R_E \approx 6371$ km denotes the Earth's radius. This angular disparity translates into a signal suppression factor of $\cos(1400/R_E) \approx 0.98$ for cross-correlations between magnetometers located in different shield rooms. Similarly, we estimate the impact of Earth's rotation over a 2000-second interval. This rotation induces a sideband with a frequency of 1 day$^{-1}$, distinct from the dark photon-dark matter peak frequency. However, this sideband is not expected to be resolved due to the frequency-domain resolution of $1/2000\,\mathrm{s}^{-1}$. For the influence on signal amplitude, a detailed analysis necessitates modeling the dark photon-dark matter wavefunctions across the entire time series. We provide a conservative estimate for the suppression factors: cos(2000 s/1 day) $\approx$ 0.9997, which is negligible.

## II. DETECTION EFFICIENCY OF DARK PHOTON

The width of the DM signal can lead to a reduction of the bin power, and the detection efficiency due to this effect should be considered. First, we consider the signal detection efficiency due to the signal power lost from binning, i.e., fractions of power falling into a single fixed bin. In a standard halo model, the velocity spread of local DPDM causes a frequency spread of $\Delta f/f_0 \approx 10^{-6}$, where $f_0$ is the Compton frequency of the DPDM. In detail, the normalized power spectrum of the DPDM signal is derived from the Maxwell velocity distribution, and the result is shown below in Fig. S2a. For example, when $f_0 = 500$ Hz, the width of DPDM signal is $\Delta f = 0.5$ mHz. On the other hand, we acquire the experimental data with a measurement time $T$, where the bin size in the frequency domain is $1/T$. For example, the measurement time is $T$ = 2000 s in our experiment and corresponds to the bin size of 0.5 mHz. We note that in this case, the DPDM signal power would experience power loss from the bin. The power spectrum of the DPDM signal derived from the Maxwell velocity distribution is used to determine empirically the fractions of power falling into a single fixed bin, where bin boundaries are systematically varied over the allowed range. To do this, we perform a numerical simulation and the frequency $f_0$ of DPDM is uniformly distributed within the allowed range.

The measurement time is set to be $T = 2000\,\mathrm{s}$, and we have simulated the detection efficiency for the DPDM signal range of 1-500 Hz. The resulting detection efficiencies ("Fraction of power detected") with respect to the frequency are shown below in Fig. S2b. As one can see, the detection efficiency is above 90% below 200 Hz and is about 75% at 500 Hz.

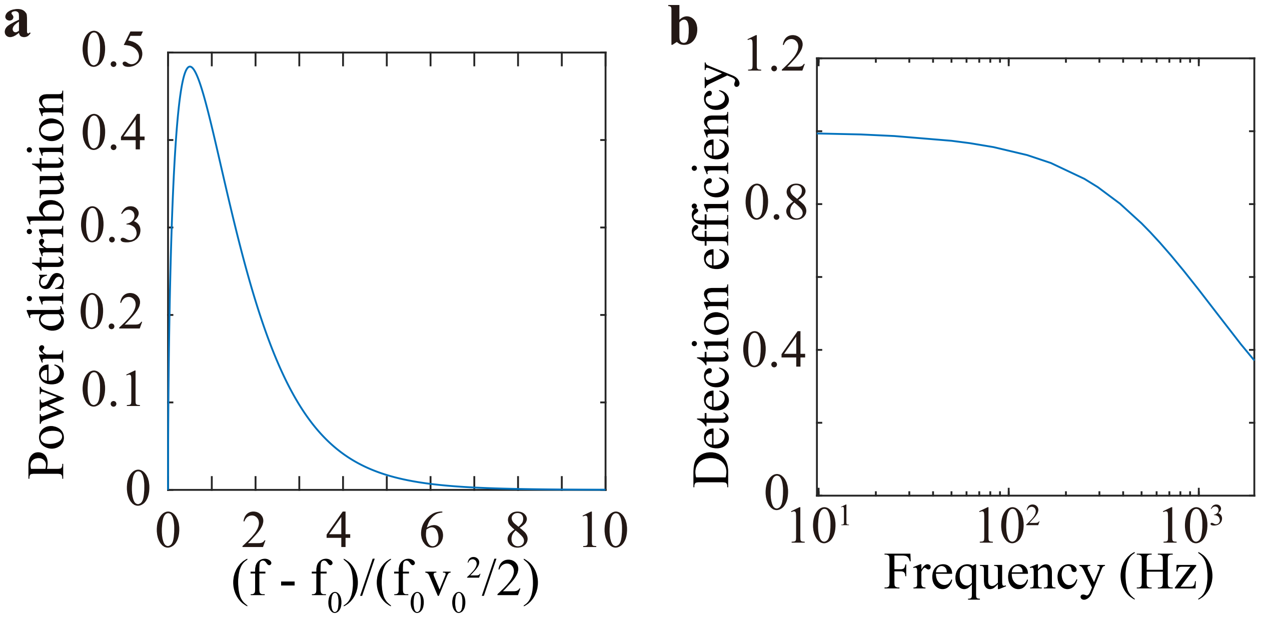


FIG. S2. **Detection efficiency of dark photon**. **a**, The normalized power spectrum of the DPDM signal is shown, with DPDM oscillation frequency $f_0$ and typical velocity of DPDM $v_0 = 230\,\mathrm{km/s}$. The normalization is chosen to make the area under the spectrum unity. **b**, The single-bin detection efficiency is shown as a function of frequency, with the bin side $\Delta f = 0.5\,\mathrm{mHz}$.

## III. QUANTUM SENSOR NETWORK

This section presents the details of our quantum sensor network for dark photon dark matter searches. The quantum sensor used in our work is atomic magnetometer that has a femtotesla-level sensitivity to magnetic field, which can be sensitive to the DPDM-induced magnetic field. The core of our DPDM searches is employing a network of 15 atomic magnetometers. In this section, we describe the basic principle of atomic magnetometers and how to correlate them into a sensor network.

We first introduce the basic principle of atomic magnetometers. Each atomic magnetometer comprises a centimeter-scale vapor cell made from pyrex glass, which contains 700 torr of $N_2$ as buffer gas, and a droplet (several miligrams) of isotopically enriched $^{87}$Rb metal. In our experiment, the atomic magnetometers operate in the spin-exchange relaxation-free (SERF) regime [6], where the spin relaxation due to the spin-exchange collision between alkali atoms can be greatly suppressed. The SERF regime can be realized by increasing the temperature of $^{87}$Rb vapor above about 150 °C and reducing external magnetic field below 10 nT. In order to operate in the SERF regime, the $^{87}$Rb vapor cell is heated to 150 °C using high-frequency AC current. All atomic magnetometers are placed inside five-layer mu-metal shield room to reduce external magnetic fields. To make $^{87}$Rb atoms become sensitive to magnetic field, it is essential to polarize and probe the $^{87}$Rb atoms. As shown in Fig. S3a, a circularly polarized laser is tuned to the D1 transition at 795 nm to polarize the $^{87}$Rb atoms and the transmission of the laser is detected with a photodiode. In a zero magnetic field, the $^{87}$Rb atomic spins align with the laser beam, and the transmission of the laser is maximized. However, a magnetic field perpendicular to the beam causes Larmor precession, rotating the magnetic moments away from alignment, leading to an absorption of the pump laser and a measurable drop in light transmission. The above discussed phenomena are zero-field resonance.

In practise, to suppress the influence of low-frequency noise of the probe beam, we apply an oscillating field along $y$ as a modulation field to converting signal to higher frequency range. The final signal is demodulated with a lock-in amplifier and is acquired with a 24-bit acquisition card. In this condition, the atomic magnetometer is sensitive to the measured field along the modulation-field direction. In our experiment, the modulation field is applied along the $y$ direction (Fig. S3a), and thus our magnetometers can be used to search for the $y$-magnetic field or DPDM-induced field along $y$.

We now introduce the quantum network of atomic magnetometers used in this work. Although a single atomic magnetometer could in principle detect the DPDM-induced mangetic field signal, it is still challenging to confidently distinguish the DPDM

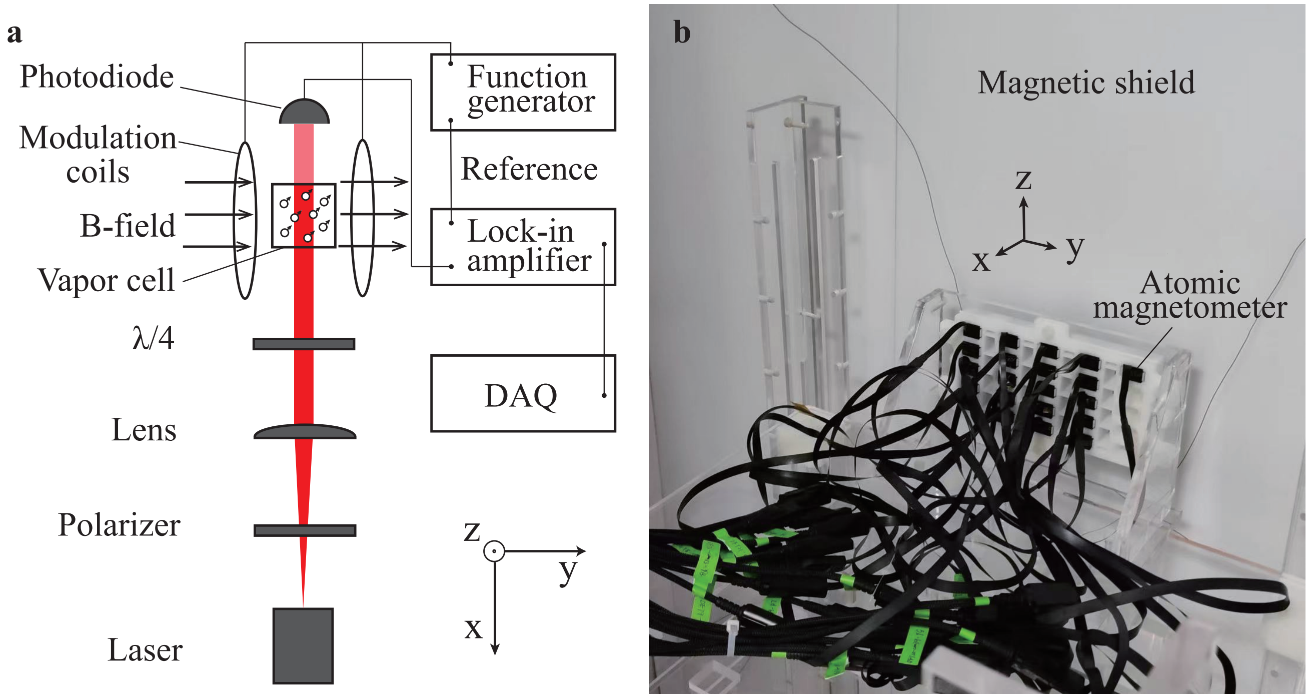


FIG. S3. **Experimental setup for dark photon dark matter (DPDM) searches**. **a**, Schematics of atomic magnetometer based on zero-field resonance. The details are present in the text. DAQ, data acquisition; $\lambda/4$, quarter-wave plate. **b**, Quantum sensor network. The Figure shown here is our Suzhou station, where there are 13 atomic magnetometers that can successfully work. The other station has 2 magnetometers and is located in Harbin, China. Such magnetometers are placed on the surface of the mu-metal shield wall. The innermost layer of the shield room is $2\times2\times2\,\mathrm{m}^3$. The sensitive directions of all magnetometers are along the $y$ axis. Here the atomic magnetometer is miniaturized into a few centimeters and its basic structure is shown in **a**.

signal from many noise sources under realistic experimental conditions. To tackle this challenge, we correlate multiple magnetometers installed in different cities to extract potential events from their correlated signals. There are 15 atomic magnetometers made by QuSpin Inc. that are compacted to a size of just a few centimeters (Fig. S3b) and realize a magnetic-field sensitivity of approximately $15\,\mathrm{fT/Hz}^{1/2}$. Such sensitive magnetometers are installed in two separate shield rooms in Harbin city and Suzhou city, China, with a distance of about 1700 km between the locations. There are 13 magnetometers installed in Suzhou station and 2 magnetometers in Harbin station. They are synchronized with the Global Positioning System (GPS) time. As discussed in Sec. I, the DPDM-induced magnetic field reaches a maximum on the source of the shield wall and is proportional to the shield size. Therefore, our DPDM search experiments should employ shield rooms as large as possible. The shield rooms in Suzhou and Harbin stations both are made from five-layer mu-metal and their innermost layer is cuboid in shape with the dimension of $2\times2\times2\ \mathrm{m}^3$. Figure S3b shows the Suzhou station, where the magnetometers are placed near the walls of the shield room with the sensitive direction along the $y$ axis.

## IV. NETWORK STATISTICS

This section presents the detailed network statistics that plays important roles in the data analysis of multiple sensors. In particular, we describe some important notations that are widely used in sensor network, for example, cross-correlation spectrum and signal-to-noise ratio (SNR) distribution. In the following, we describe such notations that are not limited in atomic magnetometers.

### A. Signal-to-noise ratio

In general, we consider that the synchronized network comprises $N_d$ detectors. For each detector, the temporal data stream $d_{m,k}=s_{m,k}+n_{m,k}$ detected by the $mth$ detector is composed of the DPDM signal $s_{m,k}$ and the measurement noise $n_{m,k}$, where $m\in[1,N_d]$ and $k\in[0,N-1]$. Here $N$ represents the total number of data points: $t_k=k\Delta_t$ refers to the time of the measurement $k$, with $\Delta_t$ being the sampling interval and $T=N\Delta_t$ being the total sampling time. The discrete Fourier transform (DFT) for the

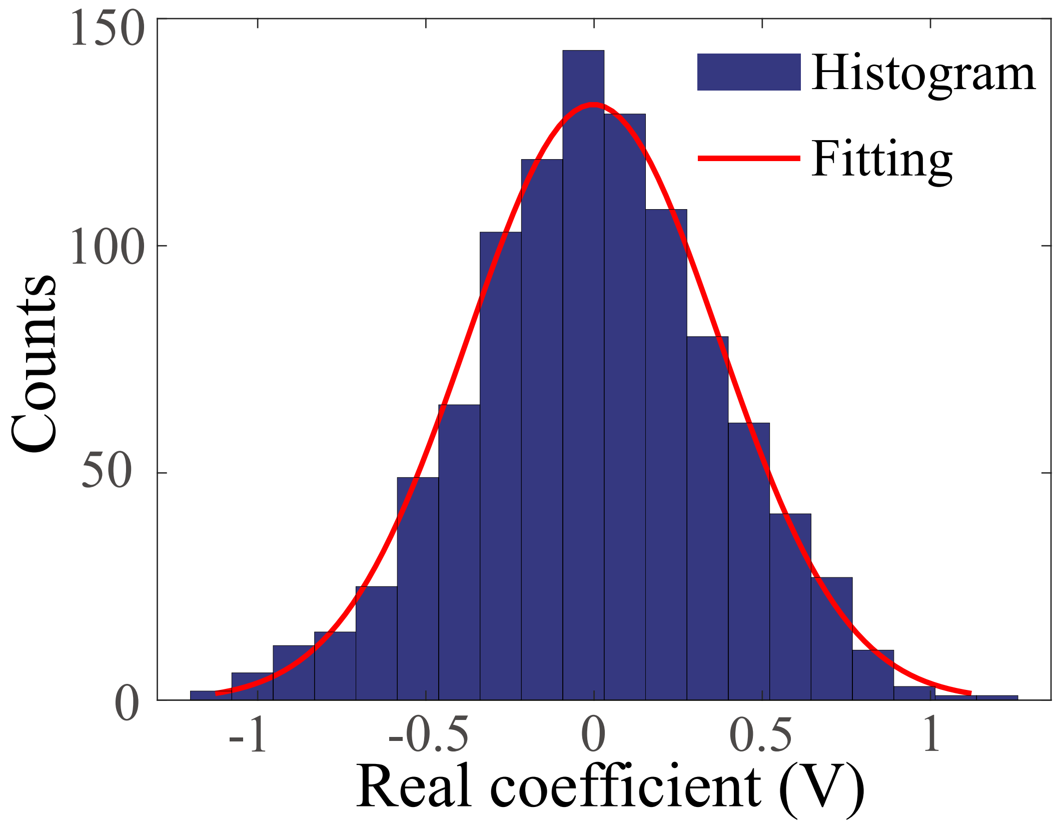


FIG. S4. **Gaussian distribution of the noise**. The noise data is collected with a center frequency of 325 Hz and a frequency width of 0.5 Hz. The real part of the FFT coefficient is used here.

$mth$ detector at frequency bin $j$ can be described by

$$z_{m,j} = \sum_{k=0}^{N-1} \exp\left(-i2\pi jk/N\right) d_{m,k}. \tag{S7}$$

It is important to study the cross-correlation spectrum that provides a powerful tool to confidently extract the DPDM signal from local measurement noise. In the frequency domain, the cross-correlation spectrum $s_{mn,j}$ for a given frequency bin $j$ between the $mth$ and $nth$ detectors is

$$s_{mn,j} = \Re\left\{\frac{z_{m,j} z_{n,j}^*}{P_{m,j} P_{n,j}}\right\}, \tag{S8}$$

where $P_{m,j}$ are taken to be the expected values for $|z_{m,j}|^2$, i.e., $P_{m,j} = \langle |z_{m,j}|^2 \rangle$. $P_{m,j}$ is estimated from neighboring, non-signal frequency bins, assuming locally flat noise (using a 50-bin running median estimate). The assumption of flat noise is validated by the Gaussian test. The distribution of the real part of $z_{m,j}$ in a frequency window of 0.5 Hz is shown in Fig. S4. The distribution is Gaussian, which can show that the noise follows the same distribution as nearby frequency bins.

Before calculating the signal-to-noise ratio, we would like to explain why we use cross-correlation rather than classical addition. In experiment, we employ 15 atomic magnetometers as detectors for dark matter. It is worth noting that the sensitivities of these detectors differ from one another for each search frequency bin. In particular, some atomic magnetometers exhibit noise peaks that are independent from one another. We show that such noise peaks can be effectively suppressed beyond the capabilities of classical addition. This improvement originates from the fact that the noise peak remains uncorrelated among the magnetometers. Moreover, we can generalize the aforementioned analysis to a general case and and derive an explicit outcome. In a general setting, the sensitivities of $N_d$ sensors are quantified by the variance of white noise, denoted as $\sigma_m^2$ where $m = 1, 2, ..., N_d$. For the classical addition method, which involves the simple summation of data from $N$ sensors and subsequent Fast Fourier Transform computation, the resulting variance can be expressed as $\sigma_{\text{classics}} = \sqrt{\sum_{m=1}^{N_d} \sigma_m^2 / N_d}$. On the other hand, in the case of correlation treatments, the variance of the signal strength can be determined as $\sigma_{\text{correlation}} = \sqrt{\frac{\sum_{m=1}^{N_d} \omega_m \sigma_m^2}{N_d \sum_{m=1}^{N_d} \omega_m}}$,

where $\omega_m = 1/P_m$ represents the weight assigned to each sensor and $P_m$ denotes the power of the $m\#$ sensor. According to a straightforward mathematical calculation, we can prove that

$$\sigma_{\text{correlation}} = \sqrt{\frac{\sum_{i=1}^{N_d} \omega_i \sigma_i^2}{N_d \sum_{i=1}^{N_d} \omega_i}} \leq \sigma_{\text{classics}} = \sqrt{\sum_{i=1}^{N_d} \sigma_i^2 / N_d},$$

where the equality holds if and only if all $\sigma_m^2$ are equal. When the sensitivities are different, the correlation is better than classical addition. However, when the sensitivities are equal, the two methods yield identical results.

We now derive the signal-to-noise ratio at a given frequency bin $j$ according to Eq. S8. The expectation value $\langle s_{mn,j} \rangle$ is zero, and however the variance is non-zero that is calculated by

$$\langle s_{mn,j}^2 \rangle = \left\langle \left( \Re \left\{ \frac{z_{m,j} z_{n,j}^*}{P_{m,j} P_{n,j}} \right\} \right)^2 \right\rangle = \frac{\langle (z_{m,j}^r)^2 (z_{n,j}^r)^2 \rangle + \langle (z_{m,j}^i)^2 (z_{n,j}^i)^2 \rangle}{P_{m,j}^2 P_{n,j}^2},$$

where the "$r$" and "$i$" superscripts refer to the real and imaginary components of the DFT. For Gaussian noise in each detector, the real and imaginary components of the DFT are taken to be independent of each other:

$$\langle (z_{m,j}^r)^2 \rangle = \langle (z_{m,j}^i)^2 \rangle = \frac{1}{2} P_{m,j}, \quad \langle (z_{n,j}^r)^2 \rangle = \langle (z_{n,j}^i)^2 \rangle = \frac{1}{2} P_{n,j}.$$

Based on the above-mentioned results, we obtain the final cross-correlation spectrum between $mth$ and $nth$ detectors

$$\langle s_{mn,j}^2 \rangle = \frac{1}{2 P_{m,j} P_{n,j}}. \tag{S9}$$

Given the total number of DFT pairs, $N_{DFT} = C_{N_d}^2$, the number of 2-combinations of a set with $N_d$, one can define the following signal strength:

$$S_j = \frac{1}{N_{DFT}} \sum_{1 \leq m < n \leq N_d} \Re \left\{ \frac{z_{m,j} z_{n,j}^*}{P_{m,j} P_{n,j}} \right\}, \tag{S10}$$

for which, in the absence of signal, the expectation value is zero and for which the variance is

$$\sigma_j^2 = \frac{1}{N_{DFT}} \left\langle \frac{1}{2 P_{m,j} P_{n,j}} \right\rangle_{N_{DFT}}, \tag{S11}$$

where $\langle \rangle_{N_{DFT}}$ denotes the average over the $N_{DFT}$ pairs. Then we define the average SNR as

$$\rho_j = \frac{S_j}{\sigma_j} = \frac{\sqrt{N_{DFT}}}{\sqrt{\left\langle \frac{1}{2 P_{m,j} P_{n,j}} \right\rangle_{N_{DFT}}}} S_j. \tag{S12}$$

In the following, we determine the $S_j$ by connecting it to the DPDM-induced magnetic field.

### B. DPDM signal and its power

As discussed in Sec. I, the DPDM produces an effective electromagnetic current inside of a shield room and then generates a magnetic field. Because our magnetometers are sensitive to the magnetic field along $y$, the DPDM-induced magnetic field along $y$ is recorded

$$\boldsymbol{B}_{\text{DP}} = B_y \sin(\omega t + \varphi) \hat{y}, \tag{S13}$$

where the oscillation frequency $\omega = m_{A'}$, and the amplitude $B_y$ of the magnetic field $\boldsymbol{B}_{\text{DP}}$ is shown in Eq. S6. The measured DPDM field corresponds to the discreted $s_{m,k}$. Because the searched mass range of dark photon is ultralight, the correlation length of DPDM ($\approx 10^{-6}\,\text{eV}/\text{m}_{\text{A}'}$ km) is much larger than the distance between Suzhou and Harbin stations. For example, for a DPDM field oscillating at frequencies $O(100)$Hz, the correlation length is $O(10^6)$ km which is much larger than the separation distance between our two stations located at Suzhou and Harbin. Hence, the detectors in such two stations experience a nearly

identical DPDM field (i.e., $s_{m,k} = s_{n,k}$), resulting in inducing strongly correlated responses. Thus, we no longer distinguish subscripts $m$.

The DPDM signal $s_{m,k}$ is measured in time domain discretely, with detecting duration $T$ and time interval $1/f_s$. Hence, the average power of DPDM signal is

$$P = \frac{1}{T}\sum_{k=0}^{N-1}|s_{m,k}|^2\frac{1}{f_s} = \frac{1}{N}\sum_{k=0}^{N-1}|s_{m,k}|^2. \tag{S14}$$

Based on Eq. S14, the average power can be estimated by using integral

$$P = \frac{1}{T}\int_0^T |B_y\sin(\omega t+\varphi)\hat{y}|^2 dt = \frac{1}{T}B_y^2\int_0^T \sin^2(\omega t+\varphi)dt = \frac{1}{2}B_y^2, \tag{S15}$$

where $T = 2\pi/\omega$ is the detecting duration. Combining Eq. S14 and Eq. S15, we have

$$\sum_{k=0}^{N-1}|s_{m,k}|^2 = \frac{N}{2}B_y^2. \tag{S16}$$

In the following, we want to determine $S_j$ for the network signal discussed in Eq. S10. According to Parseval's theorem, we can connect

$$\sum_{k=0}^{N-1}|s_{m,k}|^2 = \frac{1}{N}\sum_{j=0}^{N-1}|z_{m,j}|^2.$$

For a DPDM-induced magnetic field signal with fixed frequency, if the signal power is entirely contained in the frequency bin $j$, and suppose $mth$ and $nth$ detectors experience the same DPDM signal, we have

$$\langle z_{m,j}z_{n,j}^*\rangle = N\langle\sum_{k=0}^{N-1}|s_{m,k}|^2\rangle. \tag{S17}$$

Combining Eq. S16 and Eq. S17, we get

$$\langle z_{m,j}z_{n,j}^*\rangle = \frac{N^2}{2}B_y^2. \tag{S18}$$

Therefore, the signal strength can be rewritten in terms of the average amplitude square of the magnetic field along $y$ axis,

$$S_j = \frac{1}{N_{DFT}}\sum_{1\le m<n\le N_d}\Re\left\{\frac{z_{m,j}z_{n,j}^*}{P_{m,j}P_{n,j}}\right\} = \frac{N^2}{2}B_y^2\left\langle\frac{1}{P_{m,j}P_{n,j}}\right\rangle_{N_{DFT}}. \tag{S19}$$

### C. The connection between SNR and DPDM-induced magnetic field

The theoretical SNR for a sensor network is

$$\rho_j = \sqrt{2N_{DFT}}\sqrt{\left\langle\frac{1}{P_{m,j}P_{n,j}}\right\rangle_{N_{DFT}}}\cdot\frac{N^2}{2}B_y^2, \tag{S20}$$

which connects the measurable SNR and unknown DPDM-induced magnetic field. As discussed in the data analysis (see Sec. VI B), we can first obtain the SNR distribution from our data, then find potential DPDM candidates with high SNR beyond 95% confidence level, and then determine the potential DPDM field $B_y$. Lastly, the kinetic mixing coefficient $\varepsilon$ can be estimated from $B_y$ according to Eq. S4. The aforementioned contents are present in Sec. VI.

## V. COMMON-MODE NOISE DISCRIMINATION

This section describes how to discriminate the common-mode noise of a sensor network using cross-correlation spectra. In the main text, we find that the SNR distribution for two magnetometers located in the same shield room is asymmetric, while the SNR distribution for two magnetometers in two different shield rooms is symmetric. We explain the detailed reason as follows.

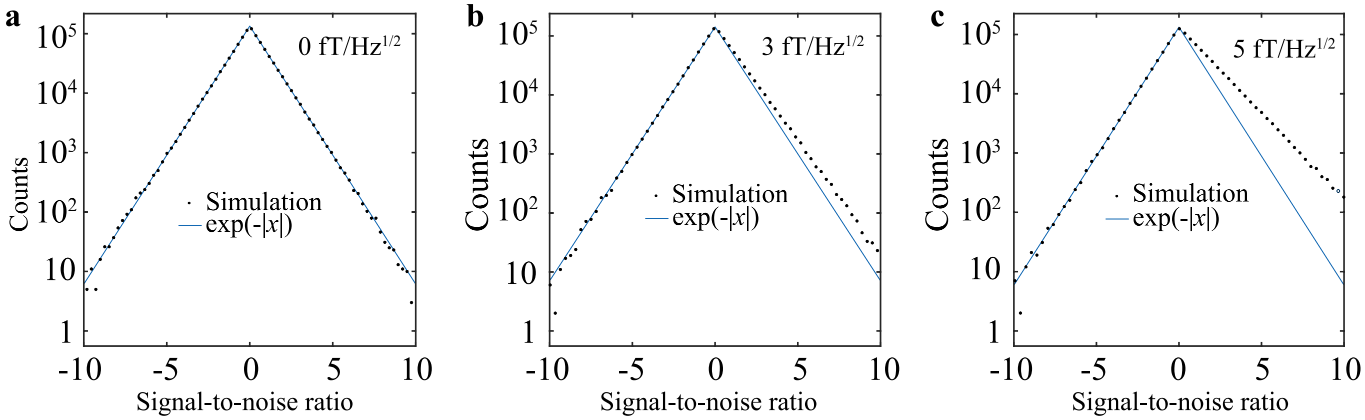


FIG. S5. **Simulated distribution of the signal-to-noise ratio (SNR) of the cross-correlation spectrum between two magnetometers**. **a**, Simulated SNR distribution without common-mode noise. The noise for two magnetometer is independent for each other. **b**, Simulated SNR distribution with the common-mode noise of 3 fT/Hz$^{1/2}$. **c**, Simulated SNR distribution with the common-mode noise of 5 fT/Hz$^{1/2}$.

It is necessary to derive the distribution of the two-point cross correlation, which is taken as

$$S = \Re\{z_1 z_2^*\}, \tag{S21}$$

where $z_1 = a_1 + ib_1$ and $z_2 = a_2 + ib_2$, and $a_1$, $b_1$, $a_2$, $b_2$ are all assumed to be Gaussian and independent. Hence, the distribution of $S$, which is calculated by $S = a_1 a_2 + b_1 b_2$, should be the convolution of

$$f(s) = \frac{1}{\pi}\int_0^\infty \frac{1}{x} e^{-\frac{1}{2}(x^2+\frac{s^2}{x^2})} dx = \frac{1}{\pi}\int_2^\infty \frac{e^{-\frac{s}{2}t}}{\sqrt{t^2-4}} dt = BesselK(0,|s|),$$

where $BesselK(0,|s|)$ is the value of the modified Bessel function of the second kind with order of $|s|$ at the point 0. The the convolution of $BesselK(0,|s|)$ is proportional to $exp(-|x|)$, and we conclude that the distribution of $s$ is proportional to $exp(-|x|)$. This indicates that the SNR distribution of two-point cross correlation should be symmetric. We perform numerical simulation and the result is shown in Fig. S9a. Our simulation result (black points) agrees with the theoretical distribution $exp(-|x|)$.

However, for two magnetometers in the same shield room, $z_1$ and $z_2$ are not independent due to the existence of common-mode magnetic noise, which is generated by the Johnson noise of the shield room. We perform numerical simulation by injecting common-mode magnetic noise, for example, 3 fT/Hz$^{1/2}$ and 5 fT/Hz$^{1/2}$ noise. The distribution of two-point cross correlation for these two sensors is no longer symmetrical as shown in Fig. S9b and c. Based on our experimental results (shown in Fig. 2 of the main text), the common-mode noise is approximately estimated to 5 fT/Hz$^{1/2}$.

## VI. DATA ANALYSIS

This section presents the detailed experimental procedure and data analysis of dark photon dark matter searches.

### A. Search experiment

Throughout the experiment, we use atomic magnetometers as DPDM detectors, which are placed near the walls of the shield rooms with the detecting direction along the horizontal tangential direction of the walls ($y$-axis) (see Fig. S3b). In order to minimize electromagnetic interference, electronic magnetometer controllers are situated outside of shield rooms and only magnetometer heads are placed inside of shield rooms. In order to operate in the spin-exchange relaxation-free regime for realizing high sensitivity, we compensate the external static magnetic field for our magnetometers as small as possible. The residual static field is typically below 0.1 nT. The bandwidth response for all magnetometers are calibrated in advances. In DPDM searches, 2000 seconds of real-time data are recorded using custom data-acquisition system, which are synchronized with the GPS time. The data sampling rate is set to be 1000 Hz. As a consequence, the detection frequency range of the magnetic field is from 1 Hz to 500 Hz, corresponding to dark-photon masses from 4.1 feV to 2.1 peV.

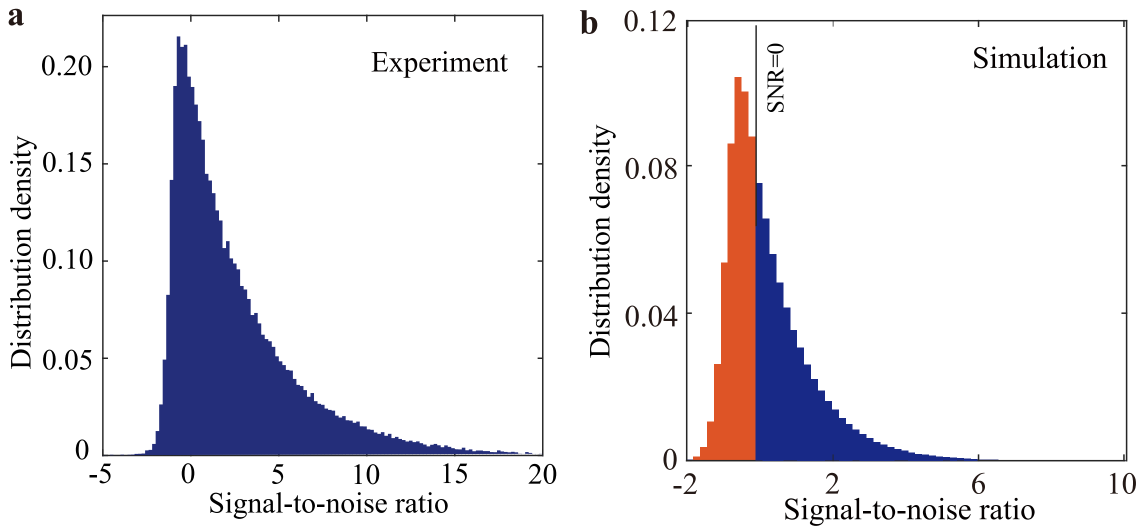


FIG. S6. **Distribution of the signal-to-noise ratio (SNR) of the network-averaged cross-correlation spectrum**. Experimental (**a**) and simulated (**b**) SNR distribution of the network-averaged cross-correlation spectrum in the frequency window of 9.1-10.1 Hz.

### B. Calculating SNR distribution

In order to find the potential DPDM candidates, we study the SNR distribution of the average cross-correlation spectrum. Firstly, we calculate the average cross-correlation spectrum using the data of 15 atomic magnetometers. For each frequency bin $j$, we can calculate the cross-correlation spectral value according to Eq. S10, and lastly obtain the complete spectrum in the range from 1-500 Hz. Secondly, we analyse the signal-to-noise ratio of the average cross-correlation spectrum at all frequency bins. The SNR $\rho_j$ at a frequency bin $j$ is calculated according to Eq. S12. We note that the SNR $\rho_j$ should satisfy a nearly identical distribution when the frequency bins are nearby. For example, in the 1 Hz-frequency window $j \in [9.1\,\text{Hz}, 10.1\,\text{Hz}]$, those SNR $\rho_j$ satisfy a nearly same distribution. As a clear illustration, the SNR distribution within the window of 9.1-10.1 Hz is shown in Fig. S6a. The distribution of the SNR is asymmetric and most of the SNR values are positive. To understand this, we perform Monte Carlo simulation, where the Gaussian noise is generated using the mean and standard deviation from our real experimental data, i.e., the background noise of atomic magnetometers. The SNR of the simulated data is calculated according to Eq. S12. The SNR distribution within the window of 9.1-10.1 Hz is shown in Fig. S6b, which agrees well with that of the experimental data.

### C. Detection threshold determination

To define the detection threshold, we use the standard $p$-value hypothesis test. Detection threshold for a hypothesis test at a frequency bin $j$ is defined by the probability to find data which are less compatible than one specific SNR (that need to be determined). We describe the details of determining the detection threshold as follows. To clearly show the procedure of determining the detection threshold, we use the case of 9.1-10.1 Hz as an example. The simulated SNR distribution is presented in Fig. S6b. The determination of detection threshold is based on the positive SNR distribution of the simulated data. This is because the sensitive directions of all magnetometers are same and the DPDM cross-correlation among different sensors should be positive. Consequently, we exclude the negative part of SNR when determining the threshold. As demonstrated in Fig. S7a, to calculate the threshold with 95% confidence level (C.L.), we integrate the distribution density of the positive SNR to obtain the threshold SNR$\approx$3.49 where the accumulated density is 95%. Compared with the detection threshold, the experimental SNR distribution ranging from 9.1-10.1 Hz is shown in Fig. S7b. The frequency bins of which the SNR exceeding the threshold are recorded as potential DPDM candidates. In order to check all frequency bins, we move the 1-Hz window with a step of 0.1 Hz and repeat the above procedure. Our initial analysis yields 318863 DPDM candidates.

After conducting the initial analysis, we find that 318863 DPDM candidates exceed the threshold with 95% C.L., which is more than the stochastic fluctuation (around 50000). In the simulation (Fig. S7b), we assume that there is no common-mode noise and technical noise in the measurements. This is primarily due to the impracticality of incorporating such noise in simulated data. The common-mode noise is due to the Johnson noise from the mu-metal shields. Different magnetometers in the same shield room measure nearly the same phase of the common-mode noise. This implies that the SNR distribution obtained by cross-correlation is biased by the positive correlation between the two magnetometers in the same shield room. Hence, the final

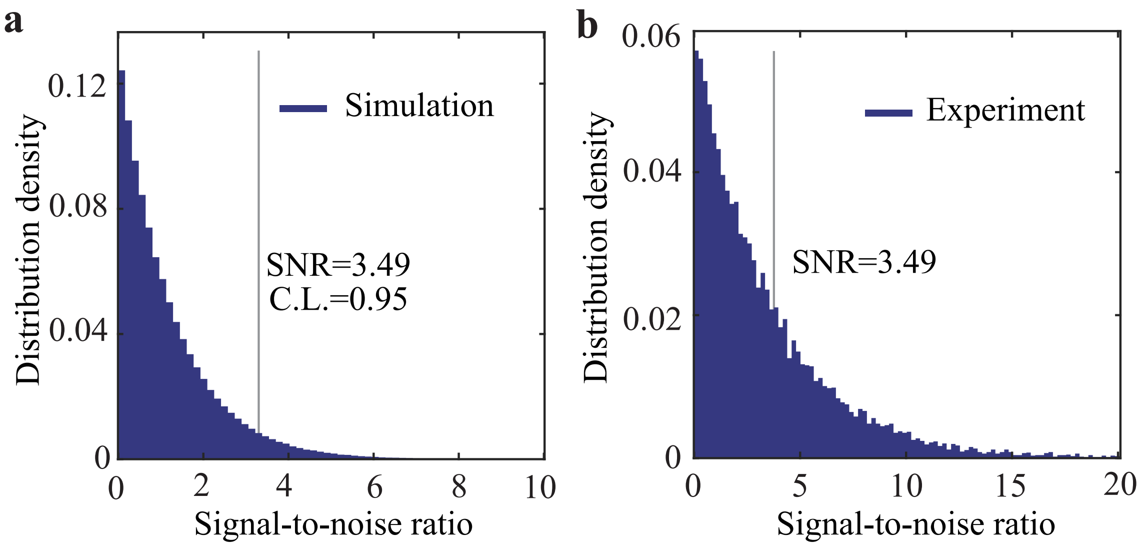


FIG. S7. **Detection threshold**. **a**, SNR distribution of the simulated data excluding the negative part, and the detection threshold is SNR≈3.49 (95% confidence level). **b**, SNR distribution of the experimental data excluding the negative part. The details are present in the text.

SNR distribution has more bins that exceed the "detection threshold" set by the expected SNR distribution.

### D. Exclusion of potential DPDM candidates

To ensure the validity of true DPDM signals and to effectively filter out false positives, it is imperative to identify the primary sources of noise and conduct a meticulous analysis of the DPDM spectral characteristics. In actual measurements, common-mode noise and technical noise are the dominant sources of the false candidates. The false signal due to common-mode noise is uncorrelated between the magnetometers in different shield rooms and can be eliminated by comparison of long-baseline networks. On the other hand, technical noise, characterized by correlated broadband noise with high SNR, may arise from factors such as sensor power supply instability. Notably, the frequency width of this broadband noise differs from that of true dark photon signals. In a standard halo model, the small but nonzero velocity spread of local dark matter causes a small frequency spread $\Delta f/f_0 \approx 10^{-6}$, where $f_0$ is the frequency of the dark photon. In our work, the searched frequency $f_0$ is below 500 Hz, and thus the intrinsic width of the dark photon signal $\Delta f$ should be smaller than 0.5 mHz. Because the width of the DFT frequency bin is 0.5 mHz in our experiment, a true dark photon signal should be included inside a frequency bin. Based on the difference in frequency width, the lagged frequency analysis can be used to distinguish a true DPDM signal from noise. During calculating the cross correlation for each two magnetometers, for example, $z_{m,j}$ and $z_{n,j}$, we lag one of the signals (i.e., $z_{n,j}$) by a large frequency difference, ensuring that no DPDM physical correlation between the two magnetometers should be expected.

In the lagged frequency analysis, a DFT frequency in one sensor is compared to a set of offset bins from other sensors such that a true dark photon signal would not contribute to a non-zero cross-correlation but for the broadband noise leads to a non-zero correlation. We choose the frequency lag $\Delta j = 10$, which is larger than the intrinsic width of the DPDM signal. For a potential signal bin $j$, the SNR $\rho_j$ is the correlation between $z_{m,j}$ and $z_{n,j+\Delta j}$. For $C_{15}^2 = 105$ pairs of cross correlation, we implement the same lag procedure, and then calculate the corresponding SNR. When the SNR is still above the detection threshold, the potential DPDM candidate should be a false signal and can be excluded. Otherwise, the SNR will be computed again with the lag changed to $\Delta j + 10$ and repeat the above procedure. The using of multiple lags is to suppress the stochastic fluctuation within the broadband noise peaks. The broadband noise may go through one lagged frequency analysis due to stochastic effect and multiple lags can reduce this effect. After our exclusion procedure, the DPDM candidates obtained in Section VI C are all excluded.

### E. Constraints on dark-photon kinetic mixing coefficient

We set constraints on dark-photon kinetic mixing coefficient because no significant DPDM candidates are found. According to Eq. S4, the measured kinetic mixing coefficient $\varepsilon$ determines the DPDM-induced magnetic field. Thus, we can obtain the constraints on $\varepsilon$ through the constraints on DPDM-induced magnetic field. To do this, we use Eq. S20 to obtain the corresponding

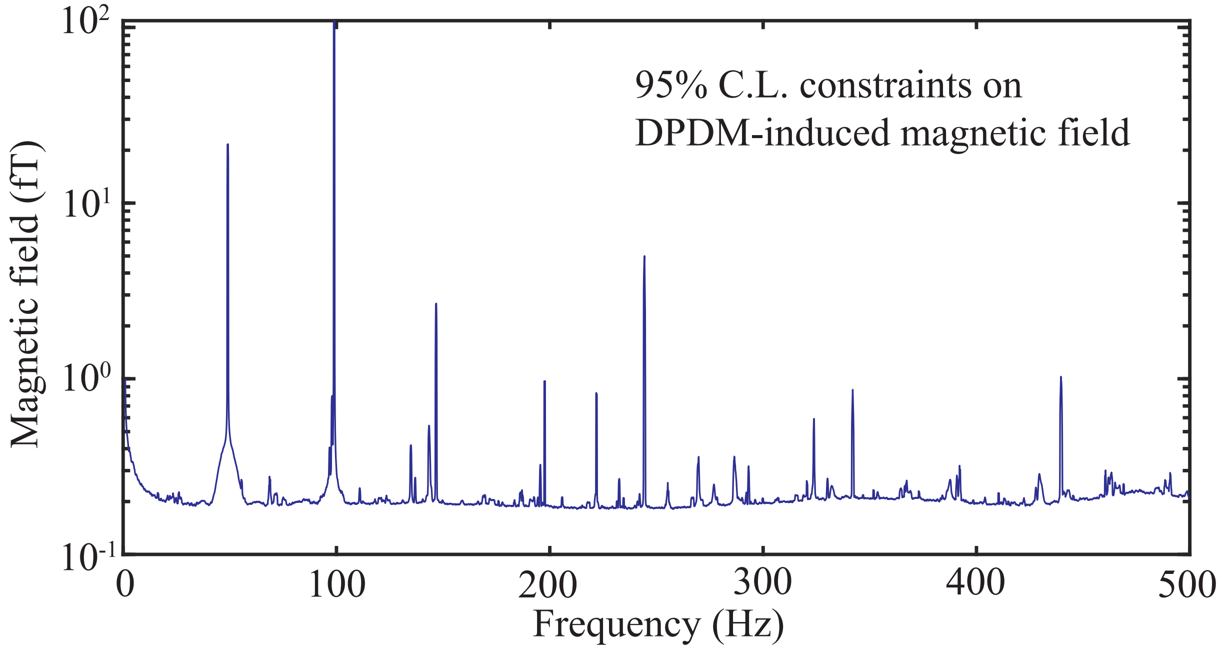


FIG. S8. **Constraints on the DPDM-induced magnetic field** (95% **C.L.**).

magnetic field from the detection threshold determined in Sec. VI C, as shown in Fig. S8. The peaks are caused by non-Gaussian noise at specific frequencies, including power noise. For these frequency bins, the standard deviation used in the Monte Carlo simulation is large, which consequently leads to a large detection threshold. Apart from these notable peaks, the constraints on the DPDM-induced magnetic field should be below 0.2 fT (95% C.L.) for most of frequency bins. The limits on the dark-photon kinetic mixing coefficient $\varepsilon$ is further obtained according to Eq. S4. For example, at the frequency 425 Hz, the magnetic field induced by the effective current of the dark photon with the mass of 1.76 peV should be smaller than 0.19 fT, and the corresponding kinetic mixing coefficient is smaller than $4.8\times10^{-6}$ (95% C.L.). The complete results of DPDM search are shown in Fig. 4b of the main text.

## VII. TESTING THE DATA ANALYSIS PROCEDURE WITH SIMULATED DPDM SIGNALS

Our data analysis procedure is tested by injecting simulated DPDM signals into our experimental data. As a demonstration, we inject a DPDM signal with the frequency of 425 Hz and with the kinetic mixing coefficient $\varepsilon = 1\times10^{-5}$ into experimental data. The simulated DPDM signal can generate an oscillating magnetic field of 0.39 fT. The corresponding average cross-correlation spectrum is shown in Fig. S9a, where the injected DPDM signal is clearly visible at 425 Hz. As shown in Fig. S9b, there is one notable point that is significantly above the 95% C.L. in the SNR distribution. According to our data analysis, we recover the DPDM-induced magnetic field as 0.41 fT, which agrees well with the injected DPDM signal. It indicates that our experiment can distinguish the simulated DPDM signal. Furthermore, we inject simulated signals with kinetic mixing coefficient $\varepsilon$ from $1\times10^{-5}$ to $1\times10^{-4}$. The recovery dark-photon kinetic mixing $\varepsilon$ are shown with blue dots in Fig. S9c, which are fitted with a linear fit (red line). The slope of the linear fit is 0.992, yielding that the kinetic mixing coefficient match the injected signals.

To check our exclusion procedure, we also checked the injected signals with "lagged frequency analysis"-signal elimination process. We find that the frequency bins with injected signals do not have any correlations with their neighbor bins, which is within expectation. Thus these injected signals pass the lagged frequency analysis and remain to be signal candidates. As we explained in Sec. VI D, "When the SNR is still above the detection threshold, the potential DPDM candidate should be a false signal and can be excluded." In this sense, the injected signal is a real DPDM signal, and the other peaks should be false signals and can be excluded.

[1] S. Chaudhuri, P. W. Graham, K. Irwin, J. Mardon, S. Rajendran, and Y. Zhao, Phys. Rev. D **92**, 075012 (2015), 1411.7382.
[2] H. An, S. Ge, W.-Q. Guo, X. Huang, J. Liu, and Z. Lu, Physical Review Letters **130**, 181001 (2023).

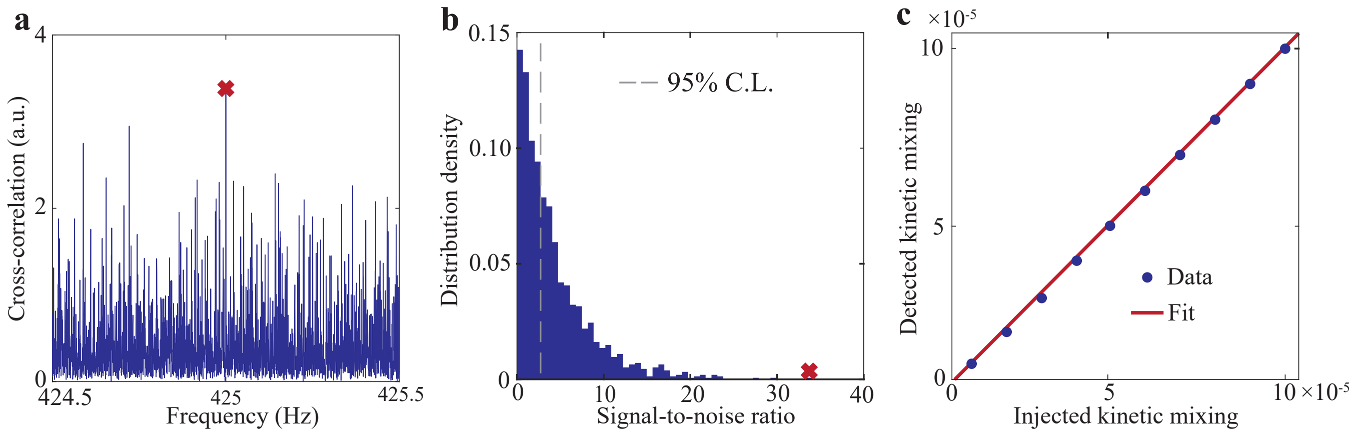


FIG. S9. **Injecting simulated dark photon signals into the experimental data**. **a**, The average cross-correlation spectrum in a frequency window ranging from 424.5 Hz to 425.5 Hz. A simulated dark photon signal (red dot) is injected with the frequency of 425 Hz and with the kinetic mixing coefficient $\varepsilon = 1 \times 10^{-5}$. **b**, The histogram of the signal-to-noise ratio within the frequency window shown in **a**. **c**, Recovery of the simulated dark photon signals, with the kinetic mixing coefficient between $1 \times 10^{-5}$ and $1 \times 10^{-4}$.


[3] J. Preskill, M. B. Wise, and F. Wilczek, Phys. Lett. B **120**, 127 (1983).

[4] L. Abbott and P. Sikivie, Phys. Lett. B **120**, 133 (1983).

[5] M. Dine and W. Fischler, Phys. Lett. B **120**, 137 (1983).

[6] I. Kominis, T. Kornack, J. Allred, and M. V. Romalis, Nature **422**, 596 (2003).
